# Electrochemical and Electrical Performances of High Energy Storage Polyaniline Electrode with Supercapattery Behavior

**DOI:** 10.3390/polym14245365

**Published:** 2022-12-08

**Authors:** Jelena Gojgić, Miloš Petrović, Branimir Jugović, Bojan Jokić, Branimir Grgur, Milica Gvozdenović

**Affiliations:** 1 Faculty of Technology and Metallurgy, University of Belgrade, Karnegijeva 4, 11120 Belgrade, Serbia; 2 Institute of Technical Sciences, Serbian Academy of Sciences and Arts, Knez Mihaljlova 35, 11000 Belgrade, Serbia; 3 Faculty of Applied Arts, University of Arts in Belgrade, Kralja Petra 4, 11000 Belgrade, Serbia

**Keywords:** galvanostatic polymerization, emeraldine state, specific power, specific energy, cycling stability

## Abstract

Polyaniline (PANI), due to its highly reversible electrochemistry with superior energy storage and delivery characteristics, is considered as an electrode material in batteries, capacitors, and hybrid systems. We used a facile electrochemical synthesis for the formation of the PANI electrode using galvanostatic polymerization of aniline on the graphite electrode at the current density of 2.0 mA cm^−2^ from the aqueous electrolyte containing 0.25 mol dm^−3^ aniline and 1.0 mol dm^−3^ H_2_SO_4_. Electrochemical and electrical characterization suggested excellent energy storage features of the PANI electrode in a three-electrode system with specific energy up to 53 Wh kg^−1^ and specific power up to 7600 W kg^−1^. After 2000 successive charge/discharge cycles at 9.5 Ag^−1^, the PANI electrode retained 95% of the initial capacity, with practically unaltered Coulombic efficiency of nearly 98%, providing a good base for future studies and practical applications.

## 1. Introduction

Intrinsically conducting polymers (ICP) are considered as perspective energy storage materials [1,2,3,4,5,6,7,8]. They can be synthesized by both chemical and electrochemical procedures; they are stable, inexpensive, ecologically acceptable, light-weighted, and have reversible electrochemistry. The classical approach to energy storage is based on batteries and electrochemical double-layer capacitors (EDC). The most important parameter that defines the type of “classical” energy storage relies on specific energy and specific power. Specific energy is related to the total amount of energy that can be stored and can be as high as 100 Whkg^−1^ in batteries. However, specific power is related to how quickly the energy can be delivered and batteries have low values of specific power, typically in the range of 0.2–1.8 kWkg^−1^ [9]. On the contrary, EDC have high specific power up to 90 kWkg^−1^ but low specific energy in the range of only a few Whkg^−1^ [9]. Pseudocapacitive electrode materials such as RuO_2_ or MnO_2_ seem to be a suitable alternative to EDC. The origin of their pseudo-capacitance, characterized by a rectangular-shaped cyclic voltammogram, comes from the Faradaic nature of the charge transport [10]. Some other newly reported carbon-based electrode materials, such as carbonized wood or various chemically activated biomass-based materials, fibers, or melanine-derived materials with superior structure, have come into focus for being eco-friendly, mechanically flexible, and environmentally stable [11,12,13]. The other alternative to EDC is the application of ICP as electrode materials. Their behavior is often treated in the literature as pseudocapacitive, although, as explained by Brousee et al. [10], the essential definition of pseudo-capacitance is not fully applicable. The energy storage mechanism in ICP is due to fast doping (oxidation)/dedoping (reduction) reactions involving anions; these reactions are characterized by battery-type cyclic voltammograms with defined peaks. However, charge/discharge curves have slopes giving a “capacitive-like” behavior. Polyaniline (PANI) is an extensively studied ICP in the field of energy storage and is denoted in the literature to be pseudocapacitive [1,3,14], with a wide range of specific and areal capacitance and both specific power and specific energy, which are strongly dependent on preparation conditions and the way these parameters are determined, namely on the potential range and discharge current. Moreover, combinations of such electrodes with “classical” battery electrodes make systems with so-called “supercapattery” behavior [8,15,16].

The specific energy is known to be dependent on the distribution of conjugation lengths in the polymer that are associated with the structure of energy bands and the number of sites that can deliver charge [14,17]. The specific power, on the other hand, is related to how quickly these energy bends are filled and emptied during potential changes and depends on many parameters, such as the thickness of the electrode, degree of crystallinity, polymer branching, etc. Therefore, it is not surprising that many parameters must be considered while creating conditions for synthesizing electrode materials. According to a review of PANI supercapacitors [1], morphology is considered crucial in achieving better performances. Soni et al. have recently achieved high specific and areal capacitance and specific energy by inducing superhydrophilicity to the electrochemical deposition of PANI on carbon paper [18]. Changing the molecular structure and intermolecular spacing achieved by the ultrathin polydopamine layer between PANI layers, as reported by Yang et al. [19], leads to PANI films with low charge transfer resistance and better electrochemical properties and stability. The main practical problem in the application of PANI is associated with volume alternation due to polymer swelling and shirking during doping (oxidation)/dedoping (reduction) of the PANI [3], and the appearance of the degradation products, which causes low cycling stability with capacitance and capacity lost. Liu et al. reported the enhancement of the cycling stability of both polypyrrole and PANI using deposition of carbonaceous shell on the polymer surface [3], which helped maintain polymer structure during long-term cycling. PANI deposition on a high surface area can lead to high specific capacitances. Most of the approaches are related to the formation of composites with carbon materials with a highly developed surface area [20,21,22,23,24,25,26,27]. Some other efficient procedures that provide better performances are also reported; for example, Chen et al. obtained polypyrrole/PANI core-shell nanostructures with super capacitive energy storage and high cyclic stability [28], whereas other authors achieved supramolecular self-assembled PANI/MXene composites with high specific capacitance and good cycling stability [29].

In this work, we used a facile galvanostatic electrochemical synthesis that enabled the polymerization of aniline on a low-cost graphite electrode to proceed at a constant rate, thus controlling the thickness and morphology of PANI, which are among the most important parameters for efficient energy storage. Moreover, we used sulfuric acid as a source of dopant ions, which means that divalent sulfate ions are shared between two polymers units to preserve electroneutrality, provoking fewer volume changes during prolonged cycling, i.e., oxidation(doping)/reduction(dedoping), thus enabling better cyclability and capacity loss. Additionally, we investigated electrochemical and electrical characteristics, including capacitance evaluated from different experimental techniques and power–energy dependence of the PANI electrode in a three-electrode arrangement which we denoted as the most suitable for the discussion of energy storage. We have pointed out that, by properly setting the working potential window, it was possible to achieve high cyclic stability with minimal capacity loss. 

## 2. Materials and Methods

### 2.1. Electrochemical Synthesis of PANI Electrode

The PANI electrode material was synthesized using electrochemical oxidative polymerization of aniline (p.a. Merck) on the cylindrically shaped graphite electrode (*A* = 0.32 cm^2^) sealed in an epoxy-made holder from the aqueous electrolyte containing 1.0 mol dm^−3^ sulfuric acid (p.a. Merck) and 0.25 mol dm^−3^ aniline at the constant current density of 2.0 mA cm^−2^, while the polymerization charge varied from 0.125 to 1.0 mAh. Prior to electrochemical polymerization, aniline was distilled in an argon atmosphere, while graphite electrodes were mechanically polished with emery papers with different grades and on polishing cloths using alumina, which was then removed ultrasonically in ethanol. Since PANI is in its doped form after galvanostatic synthesis, PANI electrodes were discharged after the synthesis procedure in the same solution, rinsed in bi-distilled water, and transferred to another electrochemical cell for further characterization. Before the electrochemical characterization experiments, new PANI electrodes were formed and held at the potential of −0.6 V for 600 s to be completely dedoped, after which the electrodes were characterized using either cyclic voltammetry or galvanostatic charge/discharge experiments. Electrochemical synthesis was performed at ambient temperature (22 ± 1 °C) in a three-compartment electrochemical cell consisting of graphite as working, Pt mesh as counter, and saturated calomel as reference electrode (SCE)

### 2.2. Characterization of PANI Electrode 

Electrochemical characterization of PANI electrodes was performed in 1.0 mol dm^−3^ sulfuric acid using cyclic voltammetry and galvanostatic cyclization experiments at different currents. Cyclic voltammetry was performed for all PANI electrodes obtained with varying charges of polymerization ranging from 0.125 to 1.0 mAh, in the potential region between −0.6 V and 1.3 V (vs. SCE), at the scan rate of 20 mV^−1^, to evaluate the charges of different PANI oxidation forms, including the degradation products, and to evaluate the specific capacitances in the broad potential window and in the emeraldine salt potential window. Cyclic voltammetry of PANI electrode synthesized with polymerization charge of 1.0 mAh was performed in the potential window between −0.6 and 0.5 V (SCE) at different scan rates ranging from 1 to 100 mVs^−1^ and used for estimation of the charge transfer control, as well as for evaluation of the maximally available capacitance. Cyclic voltammetry was also used before and after cyclization experiments to track possible changes in the nature of the charge transfer process. Galvanostatic charge/discharge experiments were performed at different current densities ranging from 1 to 50 mAcm^−2^ (0.19 to 9.5 Ag^−1^), while successive galvanostatic charge/discharge galvanostatic cycles were performed at 50 mA cm^−2^ (9.5 Ag^−1^). 

Electrochemical experiments were performed at ambient temperature (22 ± 1 °C) in a standard three-compartment electrochemical cell with PANI electrode as the working, saturated calomel electrode (SCE) as the reference, and Pt mesh as the counter electrode. The experiments were carried out using Gamry potentiostat/galvanostat 1010 E connected to a PC and equipped with electroanalysis software.

Scanning electron microscopy micrographs of a PANI electrode were taken by field emission scanning electron microscope SEM MIRA 3 TESCAN at 10 kV.

## 3. Results and Discussion

### 3.1. Electrochemical Synthesis of PANI Electrodes

**Figure 1:**
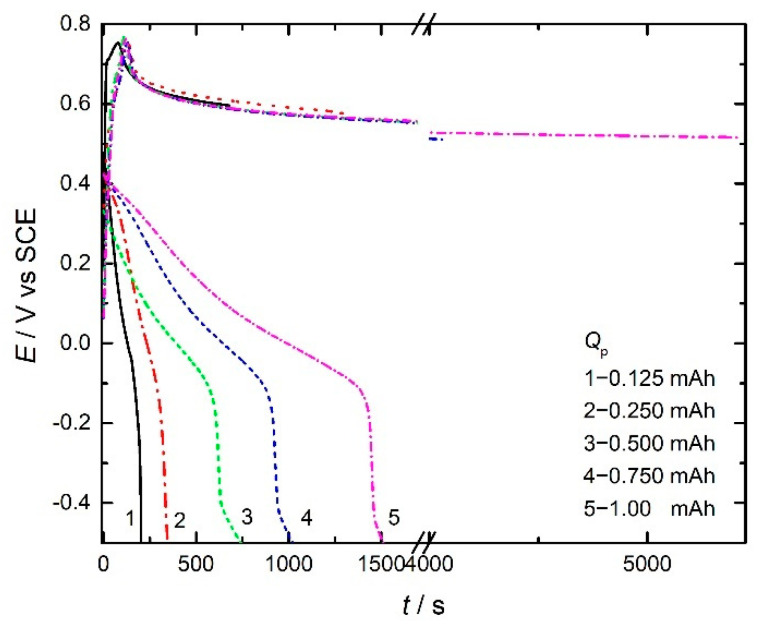
Galvanostatic curves of electrochemical polymerization of aniline in 1.0 mol dm^−3^ H_2_SO_4_ at the current density of 2.0 mA cm^−2^ with different polymerization charges, as marked in the figure, together with discharge curves of PANI electrodes with 1.0 mA cm^−2^.

Electrochemical formation of PANI electrodes with different polymerization charges ranging from 0.125 to 1.0 mAh is achieved using galvanostatic polymerization of aniline on a graphite electrode from an acidic aqueous electrolyte containing aniline (as shown in Figure 1) according to the reaction:

nANI + n(*y*/2)SO_4_^2−^ → [PANI ^*y*+^ (SO_4_^2−^)_*y*/2_]_n_ + 2nH^+^ + (2 + *y*)ne (1)

where *y* is the doping degree, i.e., the ratio between the number of charges in the polymer and the number of monomer units. Since electrochemical polymerization of aniline and growth of PANI occurs on the anode, the polymer chain is positively charged; therefore, the stochiometric quantity of dopants, i.e., sulfate ions, is necessary to preserve neutrality. 

As seen, the synthesis of PANI is characterized by a fast increase in the potential to about 0.75 V (SCE), followed by a decrease in the potential and formation of the potential plateau with nearly constant values. According to the generally accepted mechanism of oxidative radical polymerization [30], the first step refers to the formation of anilinium cation radicals through aniline oxidation at graphite anode, while the coupling of radicals with proton loss and recovery of the aromatic structure through the formation of dimmers, and subsequently oligomers, occurs in following steps. The potential plateau is related to chain propagation through further coupling of oligomers and cation radicals. The discharge curves of PANI electrodes (recorded in the same solution) have typical characteristics with a constant potential drop to about −0.2 V (SCE), after which a sharp decrease in the potential is observable, which is related to diffusion limitations. 

**Figure 2:**
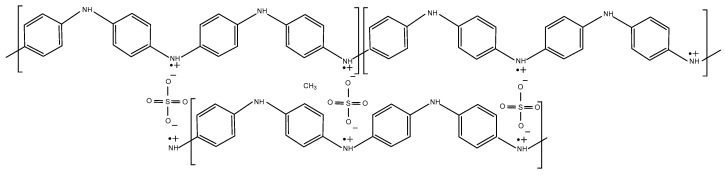
Schematic presentation of emeraldine state of PANI doped by sulfate ions.

The treatment of PANI structural behavior is practically always considered for monovalent ions’ doping; however, in this case, the doping is achieved with divalent sulfate ions. To analyze the polymerization charge and available charge for doping, the two polymer units (both consisting of four monomer units) would share two sulfate anions bridging parallel chains [6], as given schematically in Figure 2 for the emeraldine state, which is responsible for most of the available capacitance, according to recent studies [14]. 

According to Figure 2, the polymer unit is doped by two anions preserving the polaronic structure of the conductive half-oxidized emeraldine state. These anions bridge two polymer units of the parallel chains; therefore, to preserve charge neutrality, the whole polymer is doped by half of the divalent ions, i.e., *n*(*y*/2) [6]. 

Polymerization charge, *Q_p_*, can be expressed by:

(2) $$Q_{p}=I_{p}t_{p}=\left(2+y\right)neF$$

where 2neF refers to the charge necessary for polymer growth on the anode, while *yneF* refers to the charge required for doping, according to Equation (1). Assuming that the current efficiency of the polymerization process is 100%, the mass of PANI obtained with polymerization charge of *Q_p_* can be estimated relying on Faraday law according to:

(3) $$m_{\mathrm{PANI}}=\frac{I_{p}t_{p}\left[M_{\mathrm{ANI}}-2M_{H^{+}}+\left(y/2\right)M_{{\mathrm{SO}}_{4}^{2-}}\right]}{\left(2+y\right)F}$$

where *I*_p_ and *t*_p_ are polymerization current and time, respectively, while *M* stands for molar masses of aniline monomer, sulfate ion, and proton. The estimated masses of PANI obtained using different polymerization charges in the range of 0.125 to 1.0 mAh are given in Table 1. 

**Figure 3:**
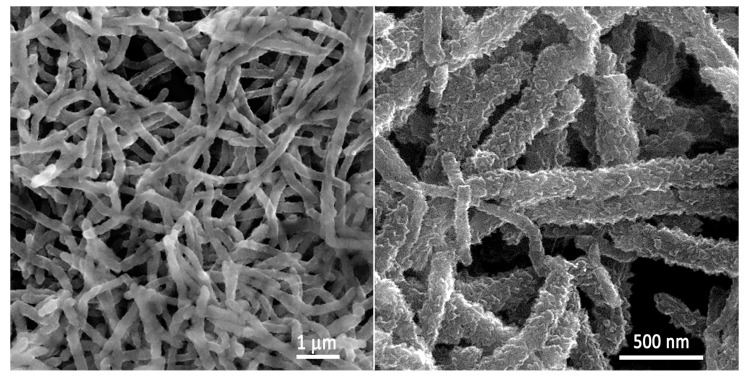
Scanning electron micrographs of an electrochemically formed PANI electrode at different magnifications, as marked in the figure.

Scanning electron micrographs of an electrochemically formed PANI electrode, taken at different magnifications, are presented in Figure 3.

As can be seen from Figure 3, PANI is uniformly deposited onto the graphite electrode, with characteristic nanosized rough fibers with a diameter between 100 and 250 nm forming a porous network with a developed specific surface suitable for efficient charge transfer.

### 3.2. Electrochemical Characterization 

**Figure 4:**
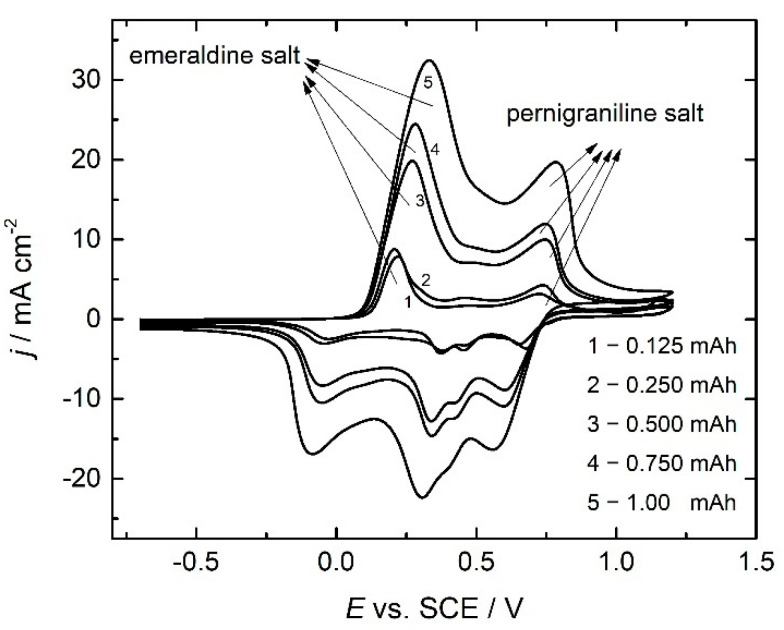
Cyclic voltammograms of PANI electrodes obtained with different polymerization charges (as marked in the figure) in 1.0 mol dm^−3^ H_2_SO_4_ at the scan rate of 20 mV s^−1^.

Cyclic voltammograms of PANI electrodes obtained with different polymerization charges (as marked in the figure) are shown together in Figure 4.

As shown in Figure 4, cyclic voltammograms of PANI electrodes have nearly the same characteristics for all polymerization charges, although the values of the peak potentials are slightly shifted to positive values (in the anodic part of voltammograms). The first anodic peak positioned at around 0.25 V is related to the doping of leucoemeraldine to half-oxidized emeraldine salt, while the second well-defined peak near the potential of 0.7 V is related to further oxidation of emeraldine salt to pernigraniline salt. Between these two well-defined peaks, there are minor peaks related to the formation of degradation products [31], primarily soluble benzoquinone and other insoluble degradation products, such as PANI strands with quinoneimine end-groups and ortho-coupled polymers remaining at the electrode surface [31]. 

**Figure 5:**
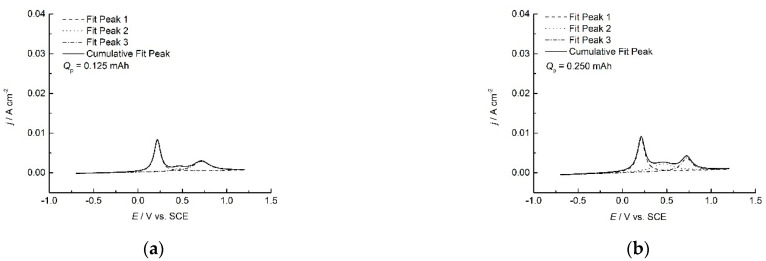

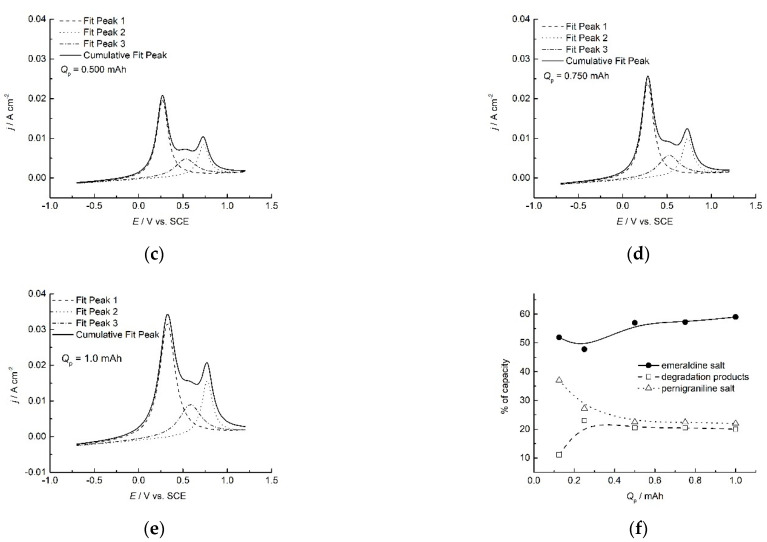
Deconvolution of cyclic voltammograms from Figure 4 for PANI electrodes obtained with different polymerization charges: (**a**–**f**) dependence of the extent of the capacity of PANI forms over polymerization charge.

To estimate the charges of different forms of PANI, the deconvolution of the anodic part of cyclic voltammograms from Figure 4 [32] was performed using Lorentzian peak fit, as presented in Figure 5, together with the dependence of the extent of these charges on the polymerization charge. 

As seen from Figure 5, it is expected that, during charge and discharge, a certain extent (around 20%) of the capacity would be permanently lost due to degradation. Therefore, it is essential to limit the working potential to avoid degradation and further transition of emeraldine to pernigraniline salt. 

Although cyclic voltammograms of PANI electrodes, as seen in Figure 4, are typical for battery electrodes with clearly observable peaks, this technique is often used in studies of supercapacitor performance of PANI for the calculation of the overall capacitance, relying on charge estimation over potential window [33]. The overall capacitance, *C*, given its definition, can be expressed as:

(4) $$C=\frac{dq}{dE}=\frac{Idt}{dE}=\frac{I}{v}$$

where, *I* stands for current and *v* for scan rate. Since the current is dependent on potential, in the defined potential window between *E*_1_ and *E*_2,_ it can be written as:

(5) $$I=\frac{1}{E_{2}-E_{1}}\int_{E_{1}}^{E_{2}}I\left(E\right)dE$$

Combining Equations (4) and (5), the capacitance calculated from cyclic voltammetry can be expressed as:

(6) $$C=\frac{1}{v}\cdot \frac{1}{E_{2\;}-E_{1}}\int_{E_{1}}^{E_{2}}I\left(E\right)dE$$

The specific capacitance, essential for practical consideration, is obtained by dividing the capacitance from Equation (6) by the mass of the deposited PANI calculated from Equation (3).

The values of specific capacitances obtained using this procedure are shown in Table 1. The given values include the specific capacitance obtained in the whole potential region, *C*_g,tot_, and the values obtained in the potential region in which no degradation occurred, namely in the potential region in which capacitance is related to the existence of half-oxidized emeraldine salt, *C*_g_. 

The values of the *C*_g_ in the potential region of existence of half-oxidized emeraldine salt are lower than those calculated for the whole potential region by less than 30% for all polymerization charges, which is consistent with the findings of Contractor and Juvekar [14], who reported that most of the available capacitance (over 70%) referred to polaron and bipolaron lattice states occurring in the potential region of emeraldine salt. Since the best value of the specific capacitance was achieved with the charge of 1.0 mAh (with a polymerization charge higher than 1.0 mAh, the active mass loss was observed), this polymerization charge was used for the formation of PANI electrode and further electrochemical characterization. 

The charge(doping)/discharge(dedoping) process of PANI electrode doped by sulfate ions is given as:

(7) $${\left[{\mathrm{PANI}}^{y^{+}}{\left({\mathrm{SO}}_{4}^{2-}\right)}_{y/2}\right]}_{n}+\left(y/2\right)ne\;\overset{{}^{\circ}\mathrm{discharge},\;\mathrm{charge}{}^{\circ}}{⇄}\;{\left[{\mathrm{PANI}}^{0}\right]}_{n}+n\left(y/2\right){\mathrm{SO}}_{4}^{2-}$$

**Figure 6:**
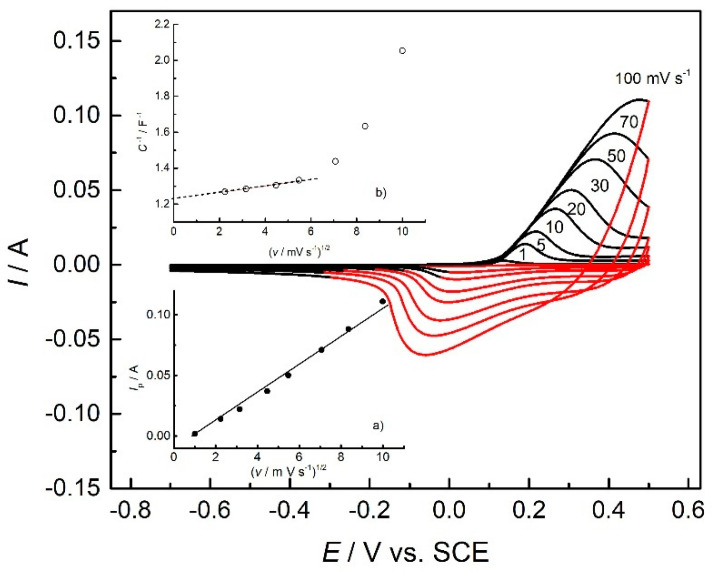
Cyclic voltammograms of the PANI electrode at different scan rates (as marked in the figure). Insert (**a**) the dependence of peak current on the square root of the scan rate and (**b**) the dependence of the reciprocal value of the capacitance on the square root of the scan rate.

To investigate the control of the charge transfer process, cyclic voltammograms with different scan rates for the freshly prepared PANI electrode were recorded and are presented in Figure 6. The anodic potential limit was set to 0.5 V to accomplish the emeraldine state and to avoid further degradation, as mentioned before. 

Based on the linear dependence of the peak current on the square root of the scan rate, it can be concluded that diffusion of sulfate ions through the pores of PANI film is a limiting factor, controlling the rate of the charge transfer process. On the other hand, the capacitance of the PANI electrode, calculated using Equation (6), decreases with the increase in the scan rate, since ion exchange is hampered through the pores of PANI. By interpolating this dependence at zero scan rate, a maximal specific capacitance of 460 F g^−1^ can be obtained.

### 3.3. Electrical Characteristics 

**Figure 7:**
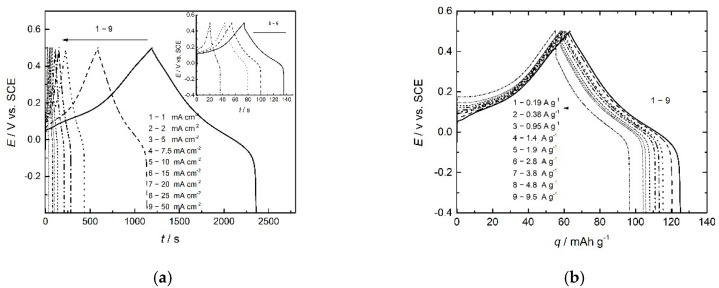
Charge/discharge curves of PANI electrode obtained with different current densities/specific currents as marked (**a**) over time, (**b**) over recalculated specific capacity.

Charge/discharge curves of PANI electrodes obtained with different current densities or specific currents and charge/discharge curves over recalculated specific capacities (mAh g^−1^) are given in Figure 7a,b, respectively. The charge potential was limited to 0.5 V to avoid degradation of PANI and further transition of emeraldine to pernigraniline salt, while the discharge limit was set to −0.4 V. 

During the discharge of the PANI electrode, sulfate ions are dedoped, leaving the polymer in a cathodic reaction, and discharge proceeds with the doping of the positively charged polymer with sulfate ions in an anodic reaction, as shown by Equation (7). 

**Figure 8:**
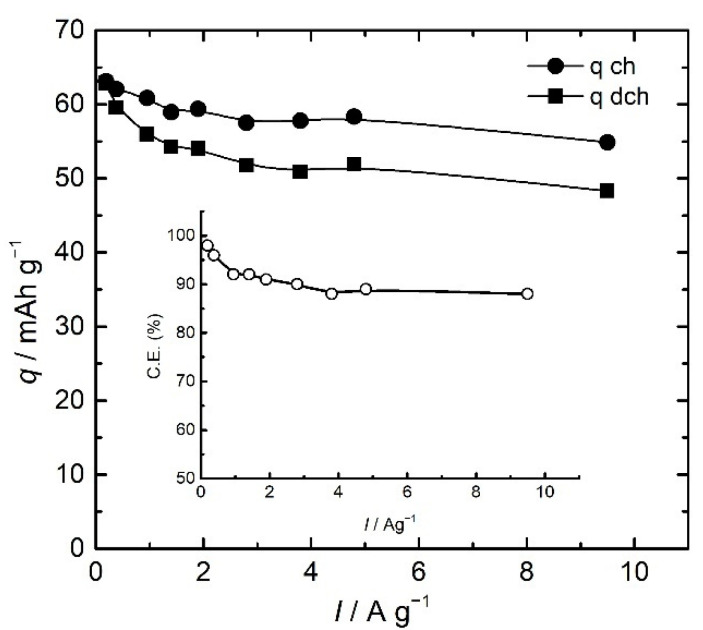
The dependence of charge, *q*_ch_, and discharge, *q*_dch_, capacity on specific current. Insert: Coulombic efficiency over specific current.

The shape of charge/discharge curves is rectangular, typical of capacitor electrodes. Both charge and discharge capacities depend on applied currents, decreasing with the increase in the current, as expected. The high values of Coulombic efficiency ranging from 98 to 90% are slightly dependent on the applied current, as seen in Figure 8.

**Figure 9:**
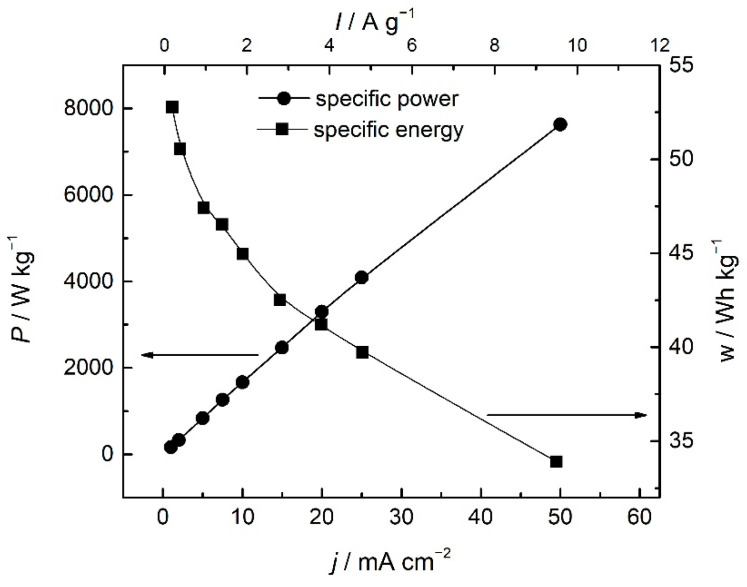
The dependence of specific power, *P*, and specific energy, *w*, of PANI vs. SCE on the discharge current.

**Figure 10:**
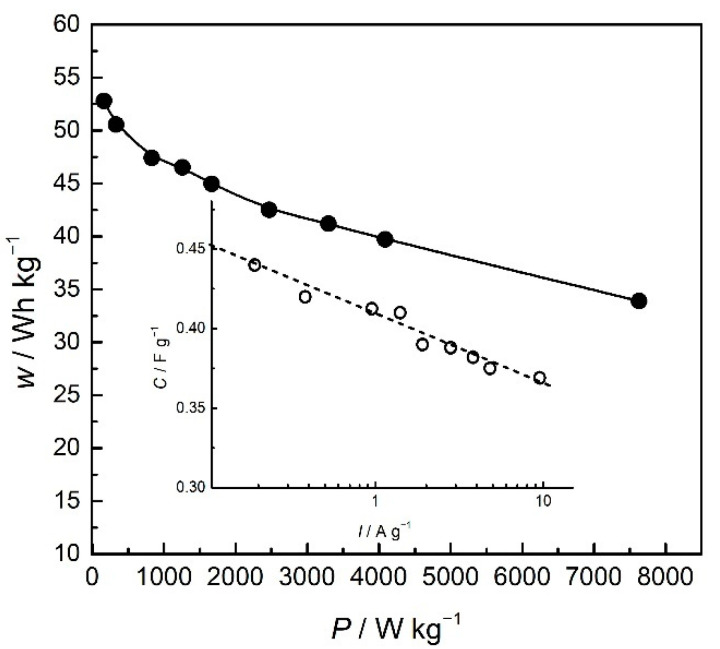
Ragone plot of PANI electrode vs. SCE. Insert: specific capacitance over specific current.

For practical considerations of an electrochemical energy storage system, the most important parameters are power and energy in their specific terms. As pointed out in the Introduction, specific energy is related to the total amount of the energy, whilst specific power is related to the speed of energy delivery. Both parameters, although characteristic of a device, can be evaluated for a single electrode in a three-electrode system and given, with respect to the reference electrode [34], by integrating discharge curves from Figure 7, as presented in Figure 9. The specific capacitance can be estimated, following the same principle, from the differential of the discharge curves (Insert of Figure 10). 

Specific energy and specific power are both dependent on discharge current, as seen in Figure 9. These values are often reported in the form of the Ragone plot (Figure 10).

Specific energy, *w* in Wh kg^−1^, at constant discharge specific current, *I* in A g^−1^, is given as:

(8) $$w=\frac{I}{3600\;sh^{-1}}\int_{0}^{t}Edt$$

and specific power can be obtained from:

(9) $$P=\frac{I}{t}\int_{0}^{t}Edt$$

while specific capacitance, *C* in F g*^−^*^1^, can be estimated from:

(10) $$C=\frac{I}{dE/dt}$$

As seen from Figure 9 and Figure 10, the specific energy of the PANI electrode (vs. SCE) decreased from 53 Wh kg^−1^ to a still very high value of 34 Wh kg^−1^ at higher specific currents. On the other hand, specific power increased, starting from the value of 200 Wkg^−1^(at 0.19 A g^−1^) to a very high value of 7600 Wkg^−1^ reported for 9.5 A g^−1^. The value of the estimated specific energy of 36 Wh kg at 1 Ag^−1^ is larger than those reported for chemically obtained nanostructured polyaniline (12.5 Whkg^−1^ at 1 Ag^−1^) [35], polyaniline composites (10.9 Whkg^−1^ at 1 Ag^−1^) [36], or substituted aniline polymers (17.5 Whkg^−1^ at 1 Ag^−1^) [6]. The value is also higher than those in the newly reported systems without intrinsically conducting polymers, such as biomass-based supercapacitors with superior capacitance. 

On the other hand, the values of the specific energy are comparable to some newly reported studies of chemically reduced graphene oxide/polyaniline composites [25] and polyaniline-loaded nanocarbon hydrogels [37]. However, specific energy is lower than the values obtained in some other systems without intrinsically conducting polymers, such as nanostructured carbonized wool-based supercapacitors, the specific energy values of which can be as high as 56.1 Wh kg^−1^ [38]. The values of the specific power reaching as high as 7600 Wkg^−1^ are obtained for 9.5 Ag^−1^ and exceed the values we have recently reported for polymer derived from substituted aniline [6] but are comparable to newly reported values for reduced graphene oxide/polyaniline composites [25]. Nevertheless, the estimated specific power of 1100 Wkg^−1^, at 1 Ag^−1^, is considerably higher than those obtained for polyaniline and some composites [35,38]. Such fast energy delivery of a PANI electrode can possibly be associated with the presence of shared sulfate ions between bridging polymer chains, which enables a faster dedoping reaction. Considering the values of specific energy and power, the PANI electrode in a three-electrode system exhibits battery behavior, especially at low currents. Simultaneously, high values of the delivered power suggest supercapacitor behavior at high currents. Therefore, the PANI electrode can be denoted as a “supercapabattery” electrode in the investigated system. 

The specific capacitance values are also decreasing over specific current (Insert of Figure 10), and the estimated maximal value of the specific capacitance from discharge curves of 452 Fg^−1^ is in excellent agreement with values obtained from cyclic voltammetry (460 Fg^−1^). Bearing in mind that polyaniline suffers from swelling and shrinking of the polymeric chains and degradation during charge/discharge, resulting in loss of the charge storage ability seen in decreased capacity retention, the PANI electrode was subjected to 2000 successive charge/discharge cycles with the potential limit for charging set to 0.5 V to avoid degradation. 

**Figure 11:**
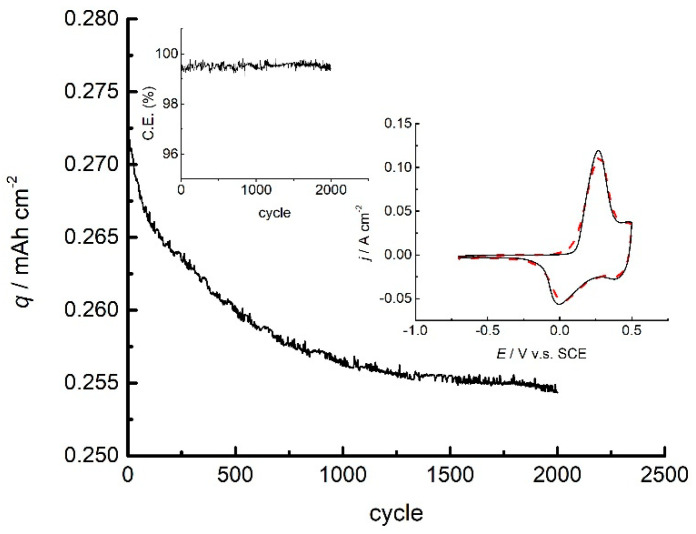
The capacity of PANI electrode over 2000 cycles of successive charge/discharge at 9.5 A g^−1^ to charge potential limit of 0.5 V. Insert: cyclic voltammograms before (full line) and after (dashed line) cycling and Coulombic efficiency over 2000 cycles.

Figure 11 shows 2000 cycles of charge/discharge of the PANI electrode, with cyclic voltammograms and Coulombic efficiency of charge/discharge over the number of cycles. 

As seen in Figure 11, the PANI electrode exhibited a slight capacity loss of only 5% during cyclization. On the other hand, the Coulombic efficiency, as high as 98%, was not altered. Moreover, the shape of cyclic voltammograms taken before and after 2000 cycles suggests no changes in the nature of the charge/discharge process, which is clearly seen in the position of the emeraldine state peak, while a negligible decrease in the peak current is related to the inevitable loss of the active electrode mass.

The excellent cyclic stability of the PANI electrode is possibly related to galvanostatic synthesis in sulfuric acid, which enables both suitable morphology and source of divalent sulfate ions bridging the polymer chains, ensuring less twisting of the chains and less volume alternation during doping and dedoping. On the other hand, proper setting of the charge potential enables minimal degradation of polyaniline electrode material. These results present a good base for further investigation that could lead to the enhancement of energy storage capability by applying this procedure in combination with other electrochemically active materials. 

## 4. Conclusions

Galvanostatic polymerization of aniline on a low-cost graphite electrode from sulfuric acid as the source of dopants was successfully used for the formation of the PANI electrode. Cyclic voltammetry experiments suggested that, during charge/discharge, some of the available charge was expected to be permanently lost by degradation and that 70% of the overall capacitance refers to the potential region of emeraldine salt, where charging potential was limited to the value of 0.5 V. Linear dependence of the peak current on the square root of scan rate indicated diffusion control of the charge transfer process. The estimated value of the maximal specific capacitance in the potential range of emeraldine state existence was 460 F g^−1^. Charge/discharge curves obtained at different current densities were used to estimate the electrical characteristics of the PANI electrode. 

Specific energy ranging between 53 Wh kg^−1^ and 34 Wh kg^−1^ and specific power between 200 W kg^−1^ and 7600 W kg^−1^ of the PANI electrode (referred vs. SCE) indicated superior energy storage properties. Moreover, the value of the maximal specific capacitance of 452 F g^−1^, derived from charge/discharge curves, agrees with the values estimated from cyclic voltammetry. The proper choice of charging potential of the PANI electrode seemed essential for achieving superior cycling characteristics, with practically constant Coulombic efficiency of 98% and minimal loss of the initial capacity. Apart from the proper choice of the charging potential, superior characteristics of the PANI electrode could also be connected to the favorable nanostructure of PANI and doping by sulfate ions that provoked less alternation of the polymer chains during charge/discharge. It is suggested that the low-cost and simple procedure of electrochemical synthesis at a constant current density, which results in a suitable morphology, together with the proper choice of the operational potential range, can lead to excellent values of both specific power and energy, as well as good cyclic stability and high Coulombic efficiency.

## Figures and Tables

**Table 1:** Mass, *m*_PANI_, specific capacitance in the whole potential region, *C*_g,tot_, and in the potential region of emeraldine salt, *C*_g_, for an electrochemically formed PANI electrode with different polymerization charges, *Q*_p,_ at the scan rate of 20 mVs^−1^.

*Q*_p_/mAh	*m*_PANI_/mg	*C*_g,tot_/F g^−1^	*C*_g_/F g^−1^	(*C*_g/_*C*_g,tot_)/%
0.125	0.22	481	378	78
0.250	0.43	290	248	85
0.500	0.87	513	375	73
0.750	1.30	436	318	73
1.00	1.73	550	404	73

## Data Availability

Upon request.

## References

[1] Eftekhari A., Li L., Yang Y. (2017). Polyaniline supercapacitors. J. Power Sources.

[2] Bryan A.M., Santino L.M., Lu Y., Acharya S., D’Arcy J.M. (2016). Conducting Polymers for Pseudocapacitive Energy Storage. Chem. Mater..

[3] Liu T., Finn L., Yu M., Wang H., Zhai T., Lu X., Tong Y., Li Y. (2014). Polyaniline and polypyrrole pseudocapacitor electrodes with excellent cycling stability. Nano Lett..

[4] Qu K., Bai Y., Gao X., Deng M. (2020). Application of poly (aniline-co-o-methoxyaniline) as energy storage material. Synth. Met..

[5] Mahdavi H., Shahalizade T. (2019). Investigation of the pseudocapacitive properties of polyaniline nanostructures obtained from scalable chemical oxidative synthesis routes. Ionics.

[6] Gvozdenović M.M., Jugović B.Z., Jokić B.M., Džunuzović E.S., Grgur B.N. (2019). Electrochemical synthesis and characterization of poly(o-toluidine) as high energy storage material. Electrochim. Acta.

[7] Hong X., Liu Y., Li Y., Wang X., Fu J., Wang X. (2020). Application progress of polyaniline, polypyrrole and polythiophene in lithium-sulfur batteries. Polymers.

[8] Han X., Xiao G., Wang Y., Chen X., Duan G., Wu Y., Gong X., Wang H. (2020). Design and fabrication of conductive polymer hydrogels and their applications in flexible supercapacitors. J. Mater. Chem. A.

[9] Yu L., Chen G.Z. (2016). Redox electrode materials for supercapatteries. J. Power Sources.

[10] Brousse T., Daniel B. (2015). To Be or Not to Be Pseudocapacitive?. J. Electrochem. Soc..

[11] Yang L., Guo X., Jin Z., Guo W., Duan G., Liu X., Li Y. (2021). Emergence of melanin-inspired supercapacitors. Nano Today.

[12] Wang Y., Li H., Yang W., Jian S., Zhang C., Duan G. (2022). One step activation by ammonium chloride toward N-doped porous carbon from camellia oleifera for supercapacitor with high specific capacitance and rate capability. Diam. Relat. Mater..

[13] Duan G., Zhao L., Chen L., Wang F., He S., Jiang S., Zhang Q. (2021). ZnCl_2_ regulated flax-based porous carbon fibers for supercapacitors with good cycling stability. New J. Chem..

[14] Contractor A.Q., Juvekar V.A. (2015). Estimation of Equilibrium Capacitance of Polyaniline Films Using Step Voltammetry. J. Electrochem. Soc..

[15] Alguail A.A., Al-Eggiely A.H., Grgur B.N. (2017). Polyaniline–lead sulfate based cell with supercapattery behavior. J. Saudi Chem. Soc..

[16] Grgur B.N., Gvozdenović M.M., Jugović B.Z., Trišović T.L. (2019). Characteristics of the citrate-based zinc–polyaniline secondary cell with supercapattery behaviour. J. Serbian Chem. Soc..

[17] Heinze J., Frontana-Uribe B.A., Ludwigs S. (2010). Electrochemistry of conducting polymers–persistent models and new concepts. Chem. Rev..

[18] Soni R., Kashyap V., Nagaraju D., Kurungot S. (2018). Realizing High Capacitance and Rate Capability in Polyaniline by Enhancing the Electrochemical Surface Area through Induction of Superhydrophilicity. ACS Appl. Mater. Interfaces.

[19] Yang M., Liu Y., Luo X., Cao Y., Gong X., Xu W. (2021). Molecular Engineering of Polyaniline with Ultrathin Polydopamine and Monolayer Graphene for All-Solid-State Flexible Microsupercapacitors. ACS Appl. Energy Mater..

[20] He Y., Han X., Du Y., Zhang B., Xu P. (2016). Heteroatom-doped carbon nanostructures derived from conjugated polymers for energy applications. Polymers.

[21] Vega-Rios A., Rentería-Baltiérrez F.Y., Hernández-Escobar C.A., Zaragoza-Contreras E.A. (2013). A new route toward graphene nanosheet/polyaniline composites using a reactive surfactant as polyaniline precursor. Synth. Met..

[22] Mitchell E., Candler J., De Souza F., Gupta R.K., Gupta B.K., Dong L.F. (2015). High performance supercapacitor based on multilayer of polyaniline and graphene oxide. Synth. Met..

[23] Tang W., Peng L., Yuan C., Wang J., Mo S., Zhao C., Yu Y., Min Y., Epstein A.J. (2015). Facile synthesis of 3D reduced graphene oxide and its polyaniline composite for super capacitor application. Synth. Met..

[24] Li K., Guo D., Chen J., Kong Y., Xue H. (2015). Oil-water interfacial synthesis of graphene-polyaniline-MnO hybrids using binary oxidant for high performance supercapacitor. Synth. Met..

[25] Zhao Z., Liu Z., Zhong Q., Qin Y., Xu A., Li W., Shi J. (2020). In Situ Synthesis of Trifluoroacetic Acid-Doped Polyaniline/Reduced Graphene Oxide Composites for High-Performance All-Solid-State Supercapacitors. ACS Appl. Energy Mater..

[26] Kim J.G., Lee D.M., Jung J.Y., Kim M.J., Khil M.-S., Jeong S.H., Kim N.D. (2021). Hybrid Polyaniline/Liquid Crystalline CNT Fiber Composite for Ultimate Flexible Supercapacitors. ACS Appl. Energy Mater..

[27] Wang Y., Hu B., Luo J., Gu Y., Liu X. (2021). Synthesis of Polyaniline@MnO_2_/Graphene Ternary Hybrid Hollow Spheres via Pickering Emulsion Polymerization for Electrochemical Supercapacitors. ACS Appl. Energy Mater..

[28] Chen S., Cheng H., Tian D., Li Q., Zhong M., Chen J., Hu C., Ji H. (2021). Controllable Synthesis, Core-Shell Nanostructures, and Supercapacitor Performance of Highly Uniform Polypyrrole/Polyaniline Nanospheres. ACS Appl. Energy Mater..

[29] Chen Z., Wang Y., Han J., Wang T., Leng Y., Wang Y., Li T., Han Y. (2020). Preparation of Polyaniline onto dl -Tartaric Acid Assembled MXene Surface as an Electrode Material for Supercapacitors. ACS Appl. Energy Mater..

[30] Zotti G., Cattarin S., Comisso N. (1987). Electrodeposition of polythiophene, polypyrrole and polyaniline by the cyclic potential sweep method. J. Electroanal. Chem. Interfacial Electrochem..

[31] Gospodinova N., Terlemezyan L. (1998). Conducting polymers prepared by oxidative polymerization: Polyaniline. Prog. Polym. Sci..

[32] Jugović B., Gvozdenović M., Stevanović J., Trišović T., Grgur B. (2009). Characterization of electrochemically synthesized PANI on graphite electrode for potential use in electrochemical power sources. Mater. Chem. Phys..

[33] Li H., Wang J., Chu Q., Wang Z., Zhang F., Wang S. (2009). Theoretical and experimental specific capacitance of polyaniline in sulfuric acid. J. Power Sources.

[34] Grgur B.N. (2021). On the Question of Energy and Power Potentials of the Electrode Materials in the Rechargeable Cells. Int. J. Electrochem. Sci..

[35] Yuan Y., Zhu W., Du G., Wang D., Zhu J., Zhu X., Pezzotti G. (2018). Electrochimica Acta Two-step method for synthesizing polyaniline with bimodal nanostructures for high performance supercapacitors. Electrochim. Acta.

[36] Li K., Liu J., Huang Y., Bu F., Xu Y. (2017). Integration of ultrathin graphene/polyaniline composite nanosheets with a robust 3D graphene framework for highly flexible all-solid-state supercapacitors with superior energy density and exceptional cycling stability. J. Mater. Chem. A.

[37] Ding J., Chen P., Chen X., Guo K. (2021). Self-Assemble Strategy to Fabricate High Polyaniline Loading Nanocarbon Hydrogels for Flexible All-Solid-State Supercapacitors. ACS Appl. Energy Mater..

[38] Yang J., Li H., He S., Du H., Liu K., Zhang C., Jiang S. (2022). Facile Electrodeposition of NiCo_2_O_4_ Nanosheets on Porous Carbonized Wood for Wood-Derived Asymmetric Supercapacitors. Polymers.

